# Seasonal Maize yield forecasting in South and East African Countries using hybrid Earth observation models

**DOI:** 10.1016/j.heliyon.2024.e33449

**Published:** 2024-06-27

**Authors:** Benson Kipkemboi Kenduiywo, Sara Miller

**Affiliations:** a International Center for Tropical Agriculture (CIAT), Kenya; b Department of Geomatic Engineering and Geospatial Information Systems, Jomo Kenyatta University of Agriculture and Technology, Nairobi, Kenya; c NASA SERVIR Science Coordination Office, Marshall Space Flight Center, 320 Sparkman Dr., Huntsville, AL 35805, USA; d Earth System Science Center, The University of Alabama in Huntsville, 320 Sparkman Dr., Huntsville, AL 35805, USA

**Keywords:** Machine learning, RHEAS, DSSAT, MODIS, Maize yield, Variable Infiltration Capacity (VIC) model, Food balance sheets

## Abstract

Climate change still adversely affects agriculture in the sub-Saharan Africa. There is need to strengthen early action to bolster livelihoods and food security. Most governments use pre- and post-harvest field surveys to capture statistics for National Food Balance Sheets (NFBS) key in food policy and economic planning. These surveys, though accurate, are costly, time consuming, and may not offer rapid yield estimates to support governments, emergency organizations, and related stakeholders to take advanced strategic decisions in the face of climate change. To help governments in Kenya (KEN), Zambia (ZMB), and Malawi (MWI) adopt digitally advanced maize yield forecasts, we developed a hybrid model based on the Regional Hydrologic Extremes Assessment System (RHEAS) and machine learning. The framework is set-up to use weather data (precipitation, temperature, and wind), simulations from RHEAS model (soil total moisture, soil temperature, solar radiation, surface temperature, net transpiration from vegetation, net evapotranspiration, and root zone soil moisture), simulations from DSSAT (leaf area index and water stress), and MODIS vegetation indices. Random Forest (RF) machine learning model emerged as the best hybrid setup for unit maize yield forecasts per administrative boundary scoring the lowest unbiased Root Mean Square Error (RMSE) of 0.16 MT/ha, 0.18 MT/ha, and 0.20 MT/ha in Malawi's Karonga district, Kenya's Homa Bay county, and Zambia's Senanga district respectively. According to relative RMSE, RF outperformed other hybrid models attaining the lowest score in all countries (ZMB: 25.96%, MWI: 28.97%, and KEN: 27.54%) followed by support vector machines (ZMB: 26.92%, MWI: 31.14%, and KEN: 29.50%), and linear regression (ZMB: 29.44%, MWI: 31.76%, and KEN: 47.00%). Lastly, the integration of VI and RHEAS information using hybrid models improved yield prediction. This information is useful for NFBS bulletins forecasts, design and certification of maize insurance contracts, and estimation of loss and damage in the advent of climate justice.

## 1 Introduction

Unpredictable weather patterns due to climate change continue to impact food production. Livelihood of farmers and those that directly and indirectly depend on agriculture have been negatively impacted by declining production during extreme weather events such as flooding or droughts. Agriculture accounts for approximately 25%-40% of nations' annual Gross Domestic Product (GDP) on average in most East African countries [8]. However, climate change, with its impact on rainfall and temperature, threatens livelihoods and food security. If climate change worsens, there will be significant impact on food security — for a projected 1.7 billion people in Africa south of the Sahara with 44% of that population being East Africans [22] — which can lead to natural resource conflicts and displacement of people seeking productive areas. There is therefore need to continuously build climate adaptation strategies especially in African countries where the majority of farming is rainfed. One of these adaptation strategies is investing in early action or warning systems in agriculture. Early action requires reliable and timely data to inform timely intervention strategies. Seasonal yield forecasts are important information for most National Food Balance Sheets (NFBS). Food balance sheets are accounting frameworks that presents a comprehensive picture of a country's food supply over a specified period [26], [18]. They support agricultural development programmes and contribute to food security and sustainable development in developing countries. This is particularly useful if such information can be provided months ahead of harvest — especially in the face of growing concerns of impacts of climate change on agricultural production — for early action or strategic decision and policy making. For instance, if a yield forecast indicates shortfalls then a country should enhance its strategic reserves while on the other hand if a bumper harvest is foreseen then a country needs to adequately prepare markets for the surplus and also ensure farmers efforts are rewarded.

Advent of prediction models using Earth Observation (EO) data has made support of early warning efforts feasible. Kenya, Malawi, and Zambia governments use agriculture pre- and post-harvest field surveys in order to capture statistics for NFBS key in food policy and economic planning. The field surveys normally involve some sampling framework and interviews that seeks to collect data on the type of crop, area planted, area harvested, quantity harvested, yield, etc [26]. Field-based surveys are however costly, time consuming, and may not offer rapid yield estimates to support governments, emergency organizations, and related stakeholders to take advanced strategic decisions. This motivated our research which explored alternative approaches using EO data to estimate yields. EO data has attractive spatial-temporal attributes of satellite remote sensing that enables rapid coverage of continents within a few days. For instance, yields can be obtained through biophysical measurement of plants, e.g., Leaf Area Index (LAI) [36], [27]. Crop yields can also be estimated from EO data through modeling.

Machine learning (ML) regression (data driven or empirical) and process based (mechanistic) EO-based models have recently been proposed as alternatives for yield prediction. To begin with, processed based models like Decision Support System for Agro-Technology (DSSAT) [24], Agricultural Production Systems Simulator (APSIM) [37], [21], WOrld FOod STudies (WOFOST) [52], STICS [5], EPIC and APEX [51], AquaCrop [46] amongst others, estimate yields by simulating crop growth process via assimilation of biophysical parameters (management and weather information) which influence growth and grain production. Developing challenges around food security, climate change, and environmental degradation has seen the advent of coupled simulation models, e.g., Regional Hydrologic Extremes Assessment System (RHEAS) [1], to address the need for sustainable agriculture in the face of resource scarcity and climate security risks [44]. RHEAS's modular approach facilitates water resources simulation using the Variable Infiltration Capacity (VIC) [31] with assimilation of remote sensing data and crop growth parameters for yield prediction using DSSAT. These mechanistic models are based on a large body of theories that cements the relationships between weather and crop yields including mechanism that influence their biophysical laws. The theoretical underpinnings establish process based models as universal but increase their complexity and demand for calibration making them less easy to use.

On the other hand, empirical approaches depend on regression of observed yields against predictors selected based on domain knowledge or feature engineering to estimate model parameters through training data that are then used to make predictions. Lischeid et al. [32] investigated Random Forest (RF) and Support Vector Machine (SVM) for crop (silage maize, winter barley, winter rapeseed, and winter wheat) yield modeling and established that their performance were similar but identification of a reliable model needed expert judgment. Studies by [45], [47] used convolutional neural network to predict wheat yields and Srivastava et al. [45] recommended use of non-linear models to effectively capture the relationship between crop yield and input data compared to linear models. For a comprehensive review of ML models application for yield prediction see [49]. Despite the growing popularity of ML models for yield prediction, the accuracy of such empirical models depends on large, reliable and orthogonal datasets while process based models require definition of many input variables [34]. Moreover, regression models perform better when predictions are made within the ranges of values observed in the training data. One way to overcome this limitation is by injecting prior knowledge using features engineered from processed based models [34]. However, notwithstanding growing research on processed based simulation, most existing studies have been implemented in developed countries [6]. Consequently, to fill these gaps we integrate the two approaches to synergize their strengths and minimize the aforementioned limitations.

Our study develops a hybrid seasonal maize yield prediction approach that can support national early action by estimating yields at county (Kenya) and district (Malawi and Zambia) level. We use the RHEAS model to generate hydrologic and crop growth features that are integrated with MODIS remote sensing indices for yield prediction using RF and SVM ML models. RF and SVM are easy to use, robust, and have been successful in many applications including gaining popularity in agronomy and environmental sciences [32]. Therefore, this study proposes a novel framework that is an improvement of the approach developed in [25] using ML models and remote sensing vegetation indices by integrating processed based features. Furthermore, the approach is developed to predict yields at sub-national level in developing countries where calibration data is sparse.

## 2 Materials and methods

### 2.1 Study area

**Figure 1:**
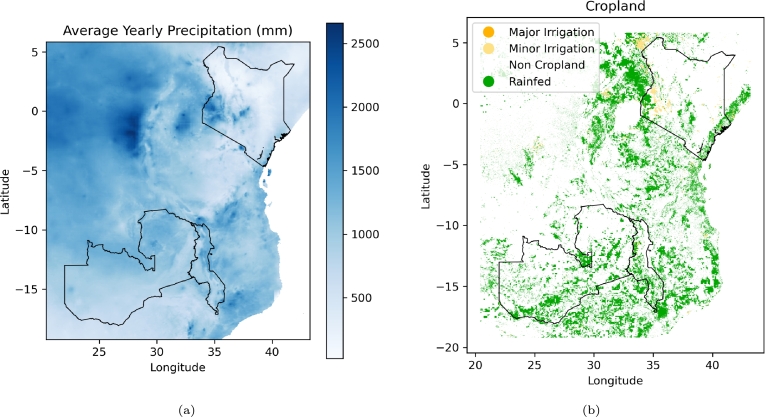
Yearly CHIRPS precipitation (a) [16] and Global Food-Support Analysis Data cropland (b) [48].

This study covers Kenya, Malawi, and Zambia (Fig. 1). Maize is a staple crop in East and Southern African countries of Malawi and Zambia, contributing significantly to the overall food security of the region. The main maize growing seasons are as follows: March–September for Kenya's main season with few parts also growing maize during October–February season; and October-April for Malawi and Zambia [15]. Average yearly precipitation varies across the region (Fig. 1a), and cropland is almost entirely rainfed (Fig. 1b). According to [15]'s country briefs, Malawi experienced a late onset of rains for 2022–2023 season with an expected worsening of food insecurity while Kenya's annual total average rainfall has declined below the long-term average for the last five years leading to mixed production prospects that is worsened by El Niño flooding in the 2023 short rain season. Lastly, the ongoing late 2023 El Nin̈o rains that has caused flooding in East Africa are also likely to cause below-average rainfall in Zambia following a three year La Nin̈a Phase. According to [12], the reduced rainfall could lead to below-average 2024 harvests in Zambia. It is evident that weather events exacerbate food insecurity and therefore necessitates investment in early action tools for better climate adaptation.

### 2.2 Methods

**Figure 2:**
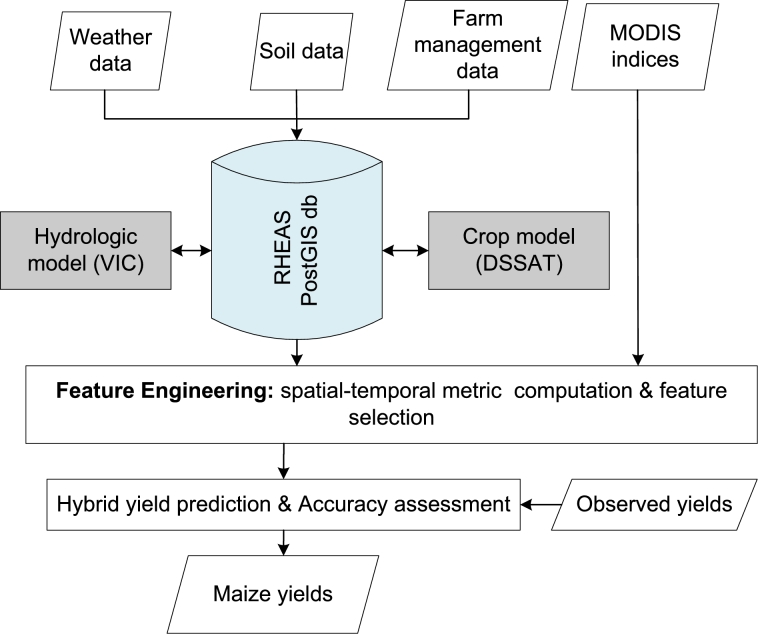
Overview of the yield prediction framework that integrates ML and RHEAS processed based model.

Fig. 2 presents a summarized prediction framework developed for maize yield prediction. Fundamental steps of the approach involved data ingestion into RHEAS VIC hydrologic model whose outputs were used in DSSAT and the hybrid models for maize yield prediction. Yields predicted using both techniques were validated against observed yield.

### 2.3 RHEAS model

RHEAS [1] is a coupled hydrologic modeling and crop simulation processed based framework which automates EO data ingestion for purposes of nowcast and/or forecasting. Hydrologic modeling is based on the VIC [31] land surface model that solves full water and energy balances while crop system simulation is implemented using the DSSAT [24]. The DSSAT model can simulate crop growth — estimating daily Leaf Area Index (DSSAT_lai) and water stress (wsgd) — and yield based on minimum input variables from VIC (soil moisture (soil_moist), precipitation (precip), wind, solar radiation, and minimum temperature (tmin) and maximum temperature (tmax), soil conditions, farm management information (maize variety, spacing, density, fertilizer type and fertilization rate).

Forcing data for RHEAS, namely rainfall, temperature, and wind, were ingested into corresponding country PostGIS databases. Rainfall data was acquired from Tropical Applications of Meteorology using Satellite data (TAMSAT; [35] approximately 4 km resolution) and CHIRPS (approx. 5 km resolution). The CHIRPS ingestion pipeline existed within RHEAS however TAMSAT ingestion framework was developed. The move to explore TAMSAT products for maize yield prediction was motivated by the findings from [33] that established it correlates better to Trans-African Hydro-Meteorological Observatory (TAHMO) in-situ data compared to CHIRPS. Macharia et al. [33] also showed that TAMSAT had less of a wet bias than CHIRPS for the region, which can inflate yield estimations. Wind, minimum and maximum temperature were obtained from the National Centers for Environmental Prediction (NCEP) (approx. 19 km spatial resolution).

Hydrologic modeling in each country was restricted to national level boundaries with a buffer of 50 km during VIC runs. VIC is a surface water and energy balance land process model that helped determine selected variables (soil total moisture (soil_moist), temperature, precipitation, soil temperature (soil_temp), solar radiation (net_short and net_long), surface temperature (surf_temp), net transpiration from vegetation (transp_veg), net evapotranspiration (evap), and root zone soil moisture (rootmoist)) that are forcing variables for the DSSAT adopted as a dynamic crop growth simulation model for maize yield forecasting.

DSSAT crop simulation requires calibration and definition of maize cultivar, type of fertilizer, soil properties [20], crop spacing, amongst other farm management information. DSSAT is a point-based system, so to estimate yields at administrative level 1 (e.g., districts in Malawi and Zambia and counties in Kenya), it was run in ensembles to select weather and soil properties within each administrative boundary. These ensemble runs were then averaged to get the yield estimations at district or county level. Calibrated maize cultivars for the region were found through a literature search and tested at administrative level 1, and the cultivar with lowest bias compared to in-situ (observed) yields was selected. Fertilizer amounts were also set in accordance with advisory from each country's Ministry of Agriculture.

### 2.4 Hybrid model

The framework was designed to use weather data (temperature and precipitation), outputs from RHEAS processed based model (soil total moisture, soil temperature, solar radiation, surface temperature, net transpiration from vegetation, net evapotranspiration, and root zone soil moisture), and MODIS vegetation indices (Gross Primary Productivity (GPP), Green Leaf Index (GLI), Structure Insensitive Pigment Index (SIPI), Normalized Pigment Chlorophyll Ratio Index (NPCRI), Moisture Stress Index (MSI), Enhanced Vegetation Index (EVI), Normalized Difference Vegetation Index (NDVI), Normalized Difference Moisture Index (NDMI), and Green Normalized difference Vegetation Index (GNDVI)). These datasets were spatially aggregated using crop masks and temporally-aggregated based on crop seasons see [25, Figure 4.].

A linear regression model (LM) alongside RF [4] and SVM [50] ML regression models were implemented based on RHEAS and MODIS vegetation indices (VI). Features with RF variance of importance above 10 were selected for maize yield prediction in each country. Two modeling configurations were designed using VI only (LM-VI, RF-VI, and SVM-VI) and based on a hybrid of RHEAS and VI features (LM-H, RF-H, and SVM-H). Relevant parameters namely radial basis kernel parameters *ϵ* and penalty *C* in SVM and number of trees, between 100–500, in RF were estimated via a grid search approach that selected parameters with the lowest mean square error. The regression model variants were compared with RHEAS maize yield simulation at county and district administration levels.

### 2.5 Model evaluation

Observed yields are essential to validate prediction models. We acquired annual average observed maize data per administrative level 1 between the years 2010–2018 (source [39]) in Kenya, 2011–2022 in Zambia, and 2012–2021 in Malawi from the ministries of agriculture after sensitization and co-development workshops on crop yield prediction using RHEAS. Maize yield estimates from the regression model configurations and RHEAS were validated against observed yields using Leave One Year Out Cross-Validation (LOYOCV) by computing percentage Relative Root Mean Square Error (RRMSE), unbiased Root Mean Square Error (ubRMSE), and Mean Bias Error (MBE) for each country and its respective counties or districts. Computation of quality metrics at county or district level is important for assessment of spatial distribution of each model's prediction. On the other hand, the LOYOCV is an iterative process that enables evaluation of a model's quality by holding yield observations of each year out of training data and using them to validate the model. This approach also allows prediction quality of the models to be temporally tested.

Assuming annual average observed yields $\overset{ˆ}{y}$ and corresponding model estimated yields *y*, the RRMSE is determined as

(1) $$\text{RRMSE}=\sqrt{\frac{\frac{1}{n}\sum_{i=1}^{n}{\left(y_{i}-\overset{ˆ}{y_{i}}\right)}^{2}}{\sum_{i=1}^{n}y_{i}^{2}}}\times 100\text{\%},$$

while the ubRMSE is

(2) $$\text{ubRMSE}=\sqrt{\frac{\sum_{i=1}^{n}{\left((y_{i}-\frac{1}{n}\sum_{i=1}^{n}y_{i})-(\overset{ˆ}{y_{i}}-\frac{1}{n}\sum_{i=1}^{n}\overset{ˆ}{y_{i}})\right)}^{2}}{n}},$$

and MBE

(3) $$\text{MBE}=\frac{\sum_{i=1}^{n}(y_{i}-\overset{ˆ}{y_{i}})}{n}$$

where *n* is the number of validation observations. The RRMSE is a dimensionless form of the root mean square error (RMSE) that indicates the overall relative accuracy of a given model compared to observed yields. It expresses prediction error as a percentage allowing for comparisons between different models. Limitations of RMSE to handle biases, for instance in estimated yields, prompted the design of ubRMSE [10]. Lastly, the MBE characterizes magnitude to which predicted yields vary from the observed ones. It shows if a model is over or under-predicting yields, e.g. positive and negative values indicate overestimation and underestimation respectively.

## 3 Results

This section summarizes the results obtained using the prediction framework described in Fig. 2. We start by presenting results of feature engineering followed by model performance and finally an example of modeled yields.

### 3.1 Feature importance

**Figure 3:**
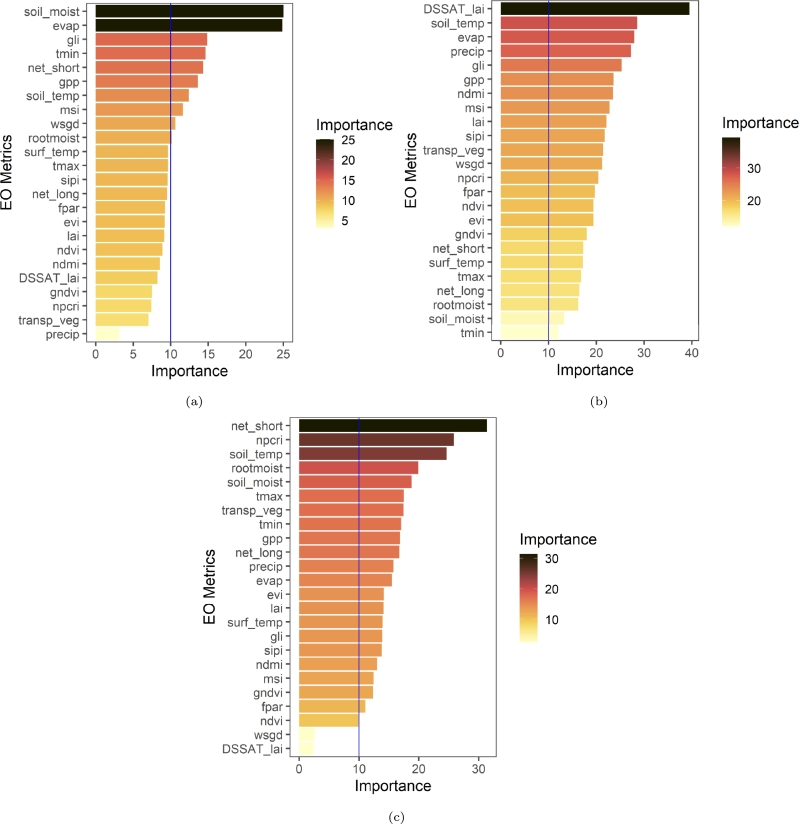
Feature importance of MODIS indices, VIC, and DSSAT features: (a) Kenya, (b) Malawi, and (c) Zambia.

Results of feature importance for each country are shown in Fig. 3. Though feature importance differs by country, all of the hybrid models include both remote sensing and model derived variables in the top three most important features. This further highlights that inclusion of physically-based modeled information is able to improve ML results. For Kenya, the most important variables are soil moisture and potential evapotranspiration. These variables are indicators of drought, signifying that moisture deficit is the greatest limitation for maize yield in the country. However, in Zambia and Malawi drought indicators are slightly lower in importance. Vegetation indices and soil temperature are higher, and for Zambia net shortwave radiation is the most important. This suggests that maize growth in Malawi and Zambia is more limited by sub-optimum temperatures and solar radiation than water availability as also portrayed by vegetation indices that are important.

### 3.2 Model performance

**Figure 4:**
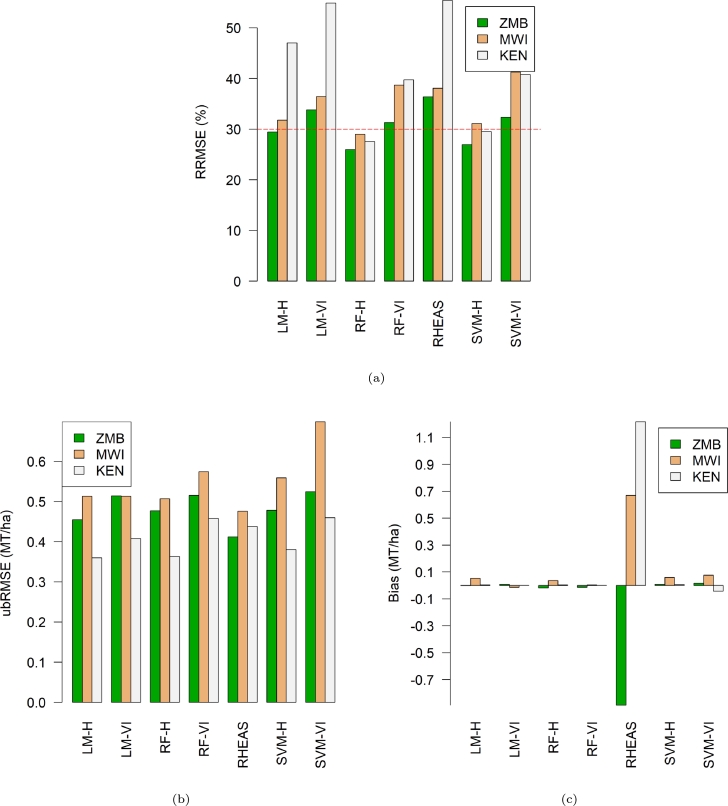
National average: (a) percentage RRMSE, (b) ubRMSE, & (c) MBE for various models based on LOYOCV. The hybrid ML models performed comparatively & had the best accuracy.

National level LOYOCV evaluation findings using RRMSE, ubRMSE, and MBE are summarized by Fig. 4. Generally, hybrid ML models perform better than regression models built with VI only and RHEAS model with regard to RRMSE (Fig. 4a). In Zambia and Malawi RHEAS yield predictions have the lowest ubRMSE and LM-H in Kenya (Fig. 4b). The RF-H configurations follow closely in all countries. A look at MBE (Fig. 4c) depicts that RHEAS over-estimate (in Malawi by 0.7 MT/ha and Kenya by 1.2 MT/ha) and under-estimate yield predictions (in Zambia by 0.9 MT/ha) by high margin compared to the other models. Two hybrid models, i.e. RF-H and LM-H, and one vegetation index model RF-VI had the least biases.

**Figure 5:**
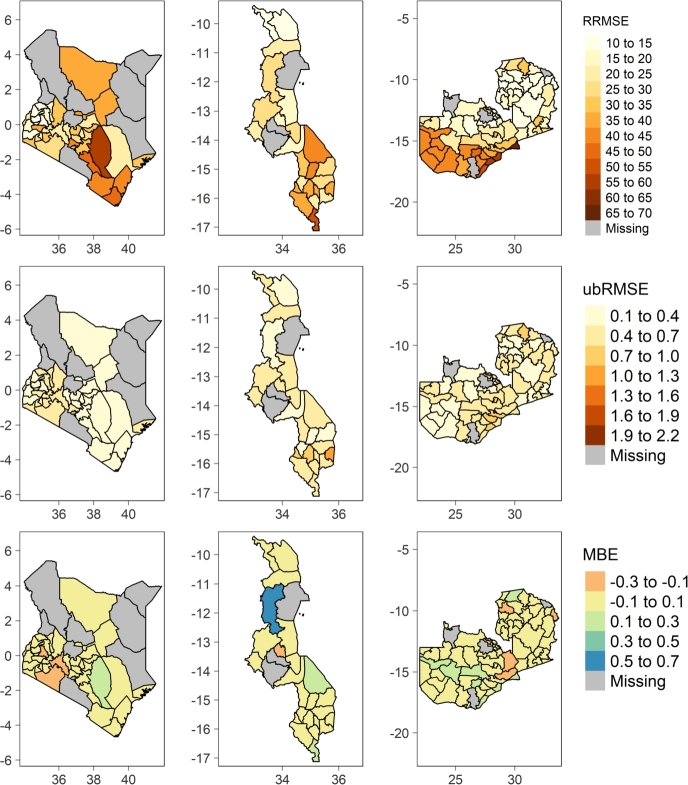
Spatial distribution of RRMSE, ubRMSE, and MBE of RF-H model in Kenya, Malawi, and Zambia respectively from left to right.

**Figure 6:**
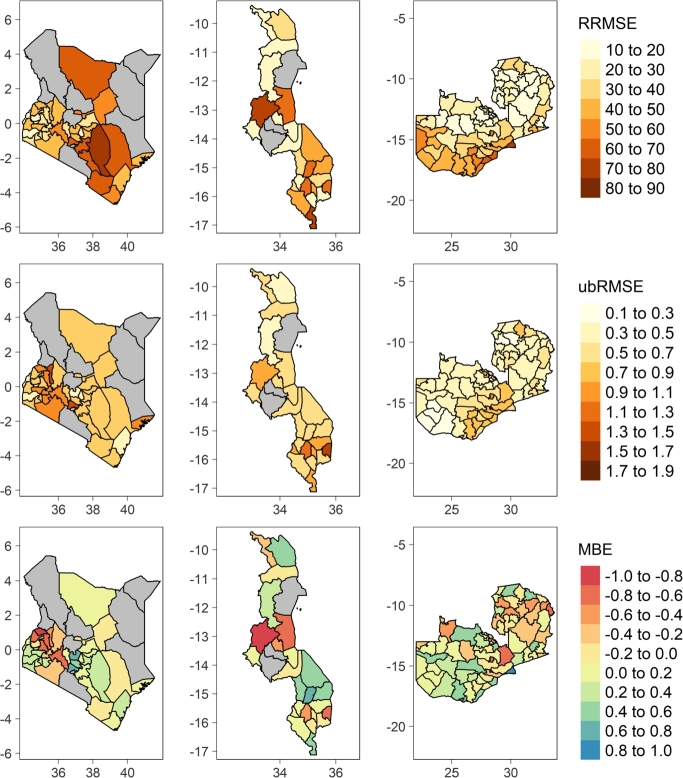
Spatial distribution of RRMSE, ubRMSE, and MBE of RF-VI model in Kenya, Malawi, and Zambia respectively from left to right.

**Figure 7:**
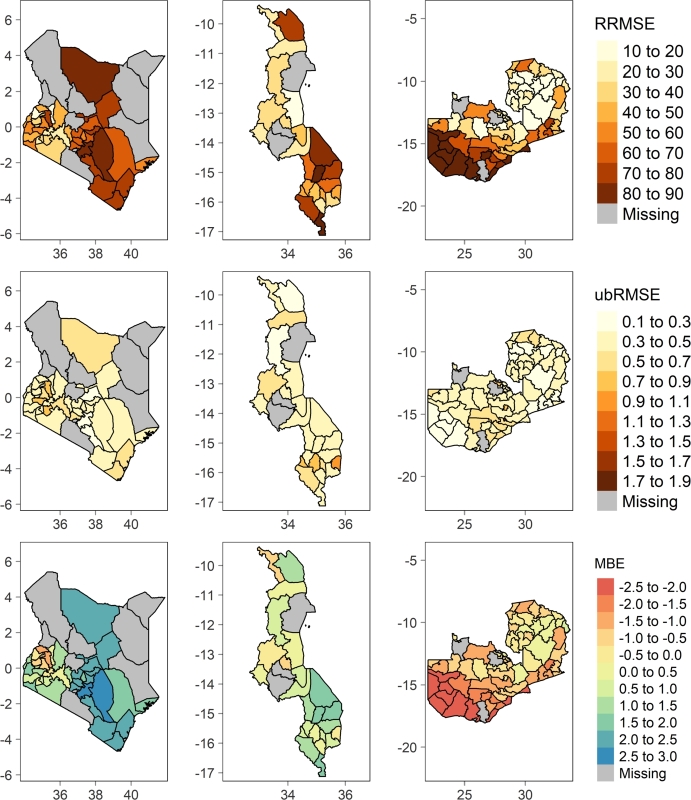
Spatial distribution of RRMSE, ubRMSE, and MBE of RHEAS model in Kenya, Malawi, and Zambia respectively from left to right.

In addition to the generalized quality metrics computed at national level, the spatial distribution of errors of each best configuration variants namely: hybrid, vegetation indices, and processed based models at county or district level were generated as illustrated by Figure 5, Figure 6, Figure 7.

In Kenya, the hybrid model configuration RF-H (Fig. 5) performs well in Kakamega county (RRMSE 9.60%, ubRMSE 0.24 MT/ha, and MBE -0.004 MT/ha) followed by other nearby counties namely: Bungoma, Vihiga, Trans Nzoia, Homa Bay, and Nandi respectively. This is reflected across the quality metrics. However, RF-H model has the lowest performance in Kitui county (RRMSE 59.10%, ubRMSE 0.26 MT/ha, and MBE 0.10 MT/ha) and nearby counties of Makueni, Kwale, and Taita Taveta in that order. These counties are characterized by semi-arid climate. Similarly in Malawi, the hybrid model still performs poorest in arid regions e.g., Nsanje district with 54.20% RRMSE, 0.43 MT/ha ubRMSE, and 0.12 MT/ha MBE. In contrast, the model performs well in Karonga district with 13.40% RRMSE, 0.16 MT/ha ubRMSE, and 0.02 MT/ha MBE. Mchinji, Rumphi, Kasungu, and Mzimba districts bordering Zambia closely trails Karonga's performance in that order. The model evidently retains the same performance trend in neighboring districts namely: Mafinga, Chama, Lundazi, and Chadiza in Zambia. Unsurprisingly, these bordering districts lie withing the same agro-ecological zones [42]. In Zambia, the model has the highest performance in Mpika district with RRMSE of 9.94%. RF-VI performance is poor in Lusaka and Luangwa including most of the southern districts e.g., Gwembe, Sinazongwe, Namwala, amongst others all which fall within the semi-arid and warm tropic agroecological zone.

The spatial distribution of errors of the vegetation indices ML model (RF-VI) is slightly pronounced compared to the hybrid model (RF-H) as depicted by Fig. 6. In Kenya, RF-VI still performs best in Kakamega county with RRMSE, ubRMSE, and MBE of 18.15%, 0.44 MT/ha, and -0.09 MT/ha respectively. However, it performs poorly with slightly higher error magnitude compared to RF-H in all semi-arid counties (Kitui, Taita Taveta, Machakos, Kwale, Kilifi, and Tana River) and arid counties (Isiolo and Marsabit). In Malawi, RF-VI performs well in Mzimba district (RRMSE of 19.29%, ubRMSE of 0.28 MT/ha, and MBE of 0.36 MT/ha). Similarly the model still performs poorly in Nsanje, being 2nd lowest, but additionally portrays the poorest performance in Kasungu district unlike in RF-H where it shows good performance. However, neighboring districts in Zambia like Mafinga, Chama, Lundazi have good accuracies with the highest being attained in Mporokoso with RRMSE of 11.02%.

RHEAS process based model shows significantly different spatial distribution of errors compared to RF-H and RF-VI Fig. 7. It has the lowest performance in Kitui, Machakos, Marsabit, Isiolo, and other lower Eastern counties in Kenya. However, it still retains good performance in the similar western counties as witnessed in RF-H and RF-VI models though with marginal differences. The same performance trend is evident in Malawi and Zambia where RHEAS performs equally poor in semi-arid regions. For instance, in Malawi it experiences very low accuracy in Nsanje and Mangochi. On the other hand in Zambia, Luangwa, Sesheke, Kalabo, and other neighboring southern districts have very low accuracy.

### 3.3 Modeled yield example

**Figure 8:**
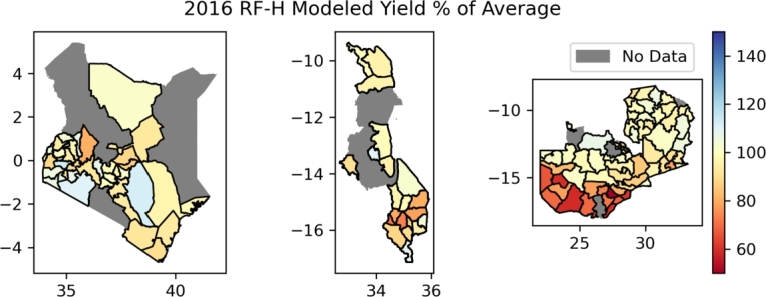
Modeled yield anomalies, expressed as a percentage of average, for 2016 using the RF-H model in Kenya, Malawi, and Zambia respectively from left to right.

To demonstrate a potential application of the RF-H model, an example of yield anomalies for the 2016 growing season is shown in Fig. 8. The 2015-2016 growing season experienced a severe drought due to El Niño in Southern Africa. In Kenya, the short rains season of 2016 experienced drought, with the rains beginning later than normal and below-average rainfall reported [40].

## 4 Discussion

### 4.1 Model performance

The aim of this paper was to develop a hybrid machine learning rapid yield forecasting model based on vegetation indices and features from RHEAS. Experiments findings indicate that integration of remote sensing vegetation indices and processed based information improved results (Fig. 3). This underscores the importance of physically simulated land surface and plant growth information in addition to conventional remote sensing plant growth observation to ML predictions. RF-H model elucidates the fact through an overall best performance in most administrative level 1 (counties in Kenya and districts in Malawi and Zambia) in all countries. It nearly eliminates the bias seen in both the RHEAS and RF-VI models while maintaining ubRMSE on average below 0.5 MT/ha. According to RRMSE classification by [30], RF-H can be considered a fair model at country level but also excels in several counties or districts. However, all the models performed poorly in semi-arid and arid areas regardless of the country.

The poor performance in arid and semi-arid areas was well highlighted by the spatial-temporal quality metrics (Figure 5, Figure 6, Figure 7). In Zambia for instance, both RHEAS and the machine learning models tend to overestimate yields for the southwestern districts. These districts have slightly lower rainfall and lower yields than those in the north. For Malawi, RHEAS generally overestimates yields most for the southern districts. In Kenya, RHEAS overestimated yields mostly for eastern counties, where there is generally less rainfall and agricultural land, and lower overall yields. Once the bias is removed, however, RHEAS is more comparable with the RF-VI and RF-H models, as the ubRMSE is on average lower for RHEAS than the other models. Spatial patterns for ubRMSE are similar between the RHEAS and RF-VI models, with higher ubRMSE in southwestern Kenya, southern Zambia, and a few districts distributed throughout Malawi.

Despite low performance in arid and semi-arid areas, yield predictions over a growing season that experienced a severe drought (Fig. 8) does not show significant deficits. In 2015-2016 growing season, Southern Africa experienced a severe drought due to El Niño. This also affected the short rains season of 2016 in Kenya with the rains beginning later than normal and below-average rainfall reported [40]. Since the short rains are one of two cropping seasons in Kenya, it is reasonable that the modeled yields for 2016 do not show deficits as strongly as in Malawi and Zambia. For Malawi and Zambia, the greatest deficit of modeled yields occurs in the south of each country. This is in agreement with results from the GEOGLAM Early Warning Crop Monitor at the end of the 2016 season [17], as well as projected food security outcomes from FEWS NET reports [13], [14].

### 4.2 Comparison with existing models

The hybrid approach presented here aims to fill several gaps in existing studies for maize yield estimates in sub-Saharan Africa. Firstly, this model provides maize yield at an administrative level 1 (counties or districts). Due to the amount of information required for physically based models, most existing studies for the region are at a specific location rather than covering a whole county or district [28] [41] [19] [7]. Some EO-based models exist for the region, but either provide yield estimates at the country level, or do not currently cover all countries of interest [2] [3] [29]. To the best of our knowledge, none of the existing ML models at sub-national levels integrate non-EO datasets such as solar radiation, which was found to be an important feature in this study. Lee et al. [29] presents an EO-based ML model, which overlaps with our study area in Malawi and Kenya. Their end of season yield estimates have a MAPE of around 0.3 for Malawi and 0.5 for Kenya, somewhat comparable to the RRMSE of 25–30% by the RF-H model of this study. Other maize models at county level for Eastern Africa also found an overestimation of modeled yields in low-yielding regions in Kenya and Malawi [29] [9].

### 4.3 Limitations and future work

There are a few potential reasons for over-estimation of yields in the models. Firstly, during co-development workshops in Zambia it was noted that the ministry of agriculture official maize yield statistics did not consider green maize harvested early for sale in markets, those used for silage, and for commercial seed production. Secondly, most arid and semi-arid counties experience total crop failure owing to frequent severe droughts [38], [11]. Machine learning and RHEAS process based model were not able to capture the crop failure events with the impact being pronounced in RHEAS. However, it is notable that integration of RHEAS hydrologic information through VIC and plant growth information through DSSAT significantly improved the machine learning predictions in arid and semi-arid lands (ASALs). The importance of coupling of hydrologic features has been noted using other models like APSIM by [43]. Nonetheless, predictions from any of processed based models can be good, but they suffer from complexity in an operational setting because many input datasets and assumptions must be managed [23]. Factors such as pest infestation, post-harvest losses, or variable management practices over different areas could not be accounted for. Lastly, RHEAS predictions are based on ensemble samples of weather and soil information. It is possible that these samples were not enough to capture representative indicators to estimate important parameters such as soil moisture hence leading to the high bias from RHEAS model in ASALs. There are also areas under maize irrigation in the ASALs, e.g., the Galana-Kulalu Irrigation Project and Holo-Holo Irrigation scheme in Tana River County. Areas under irrigation schemes are not well modeled because of reliance on weather information for prediction, and rainfed conditions were assumed since the vast majority of croplands are rainfed. Nonetheless, it was interesting to note that the hybrid model was also able to make accurate predictions in areas dominated by small-scale farmers like the western regions of Kenya. Small-scale farmers are key contributors to food security and therefore accounting for their production accurately is important for situational analysis. Both the hybrid and RHEAS models still perform fairly overall, and therefore are useful in supporting food balance sheets and country briefs. Future studies will explore the possibility of using seasonal forecasts in the hybrid model to provide advanced maize yield estimates.

## 5 Conclusion and outlook

The hybrid models designed in this study have demonstrated the ability of estimating unit maize yields. Despite challenges associated with operational complexity of processed based models, physically simulated information generated from them plays a significant role in ML prediction accuracy. Moreover, if such models are well operationalized—in terms of input data and assumptions—they can accurately simulate physical plant growth conditions supplementing pure empirical ML models that rely heavily on observed field training data. Therefore, hybrid approaches can be adopted for rapid assessment saving on field-based surveys which are time consuming and costly. In addition, with the advent of climate justice through administration of loss and damage fund, such tools can play a critical role in assessing losses and consequently compensation to farmers impacted by climate extreme events. Moreover, one component of adaption to climate change involves investment in early warning tools for which one potential framework has been demonstrated by this study. Our follow up study will explore how to integrate VI with RHEAS's advance forecasts to estimate maize yields given the success of this study.

## CRediT authorship contribution statement

**Benson Kipkemboi Kenduiywo:** Writing – original draft, Methodology, Data curation, Conceptualization. **Sara Miller:** Writing – original draft, Conceptualization.

## Declaration of Competing Interest

The authors declare that they have no known competing financial interests or personal relationships that could have appeared to influence the work reported in this paper.

## Data Availability

Remote sensing data used in this study are publicly accessible. Observed maize yields in Kenya can be accessed via KilimoSTAT (https://statistics.kilimo.go.ke) while those in Malawi and Zambia requires official request from relevant ministries.
